# Statistical Modeling of Large-Scale Signal Path Loss in Underwater Acoustic Networks

**DOI:** 10.3390/s130202279

**Published:** 2013-02-08

**Authors:** Jesús Llor, Manuel Perez Malumbres

**Affiliations:** Physics and Computer Engineering Department, Miguel Hernandez University, Ave. Universidad S/N, Ed. Alcudia, 03202 Elche, Alicante, Spain; E-Mail: jesusllor@gmail.com

**Keywords:** wireless sensor networks, underwater acoustic communications, acoustic propagation, statistical modeling, network planning

## Abstract

In an underwater acoustic channel, the propagation conditions are known to vary in time, causing the deviation of the received signal strength from the nominal value predicted by a deterministic propagation model. To facilitate a large-scale system design in such conditions (e.g., power allocation), we have developed a statistical propagation model in which the transmission loss is treated as a random variable. By applying repetitive computation to the acoustic field, using ray tracing for a set of varying environmental conditions (surface height, wave activity, small node displacements around nominal locations, *etc.*), an ensemble of transmission losses is compiled and later used to infer the statistical model parameters. A reasonable agreement is found with log-normal distribution, whose mean obeys a log-distance increases, and whose variance appears to be constant for a certain range of inter-node distances in a given deployment location. The statistical model is deemed useful for higher-level system planning, where simulation is needed to assess the performance of candidate network protocols under various resource allocation policies, *i.e.*, to determine the transmit power and bandwidth allocation necessary to achieve a desired level of performance (connectivity, throughput, reliability, *etc.*).

## 1. Introduction

The growing need for ocean observation and remote sensing has recently motivated a surge of research publications as well as several experimental efforts (e.g., [1]) in the area of underwater acoustic networks (UANs). It is crucial to UAN development to understand the propagation conditions that define the time-varying and location-sensitive acoustic environment, not only from the viewpoint of small-scale, rapid signal fluctuations that affect the performance of the physical layer techniques, but also from the viewpoint of large-scale, slow fluctuations of the received signal power that affect the performance of higher network layers.

This fact has been gaining recognition in the research community, leading to increased awareness about the need for network simulators that take into account the physics of acoustic propagation [1–4]. As a result, the first publicly available acoustic network simulators have emerged [5], and more are likely to come. One of the challenges in the design of underwater acoustic networks is the power allocation across different network nodes. This task is exacerbated by the spatial and temporal variation of the large-scale transmission loss (TL), and the lack of statistical models that capture these apparently random phenomena.

While it is well known from field experiments that the received power varies in time around the nominal value predicted by a deterministic propagation model, little is known about the statistical nature of these variations. Literature on this topic is scarce; however, several recent references indicate that the received signal strength obeys a log-normal distribution (e.g., [6–8]). A good system design has to budget for signal strength variations in order to ensure a desired level of network performance (*i.e.*, connectivity), and the budgeting task can be made much easier if the statistics of the underlying process are known.

**Figure 1.**
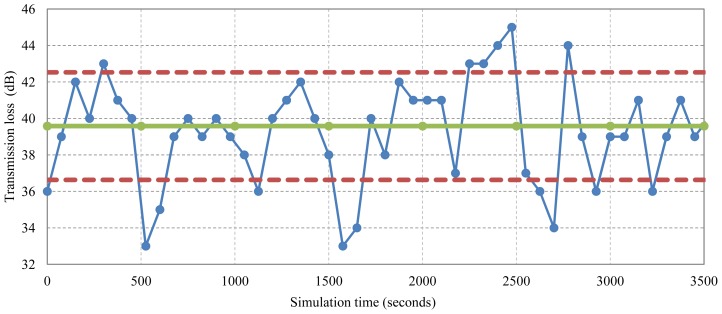
An ensemble of transmission losses calculated by the Bellhop model. Solid line indicates the average calculated over the total run time. Dashed lines indicate the values of one standard deviation σ.

In this work, we analyze those random variations in the large-scale transmission loss that are mainly governed by environmental factors, such as surface activity (waves) for a particular network scenario. We begin by employing a prediction model based on the Bellhop ray tracing tool [9]. Such a deterministic model provides accurate results for a specific geometry of the system, but does not reflect the variations that occur as the geometry changes slightly, due to either surface motion or transmitter/receiver motion. Figure 1 illustrates this situation for a point-to-point underwater acoustic link. It shows an ensemble of transmission losses calculated by the Bellhop model for a set of varying surface conditions, each one slightly different from the nominal.

While it is possible, in principle, to run a deterministic propagation model for a large number of different surface conditions, the underlying computational demands are high. In a large network, it is ineffective, and possibly not even feasible, to run a complex prediction model for each packet transmission. A statistical prediction model then becomes necessary.

The goal of our work is to employ an existing deterministic prediction model (DPM), such as the ray tracer [10], to generate an ensemble of channel responses corresponding to varying propagation conditions in a given network scenario. Using the so-obtained values, we then conduct a statistical analysis to obtain the probability density function (pdf) of the large-scale transmission loss. The result is a detailed statistical prediction model (SPM), like the ones proposed in [11,12], that is easy to employ for network design and analysis. Then, the SPM model would be easily integrated in a network simulation tool to reproduce the acoustic signal attenuation map of the network scenario, resulting in a significant reduction of the overall simulation complexity with acceptable prediction accuracy when compared to the one obtained through the deterministic prediction model. As a consequence, the SPM model enables computationally-efficient inclusion of fading effects into the network simulator. Namely, to assess the average system performance, a network operation has to be simulated over a large set of channel realizations (*i.e.*, varying surface conditions).

Whereas repeated computation of the ray trace for different hops that each of the data packets traverses in a given network may be computationally prohibitive, statistical modeling requires only a single call to the Gaussian random generator for each packet transmission. Thus, the overall simulation time is considerably reduced, allowing a system designer to freely experiment with different network protocols and resource allocation strategies in an efficient manner. So, the ultimate goal is to choose the best upper-layer protocol suite and to relate the necessary system resources (power, bandwidth) to the propagation conditions, *i.e.*, to the statistical parameters of the transmission loss.

**Figure 2.**
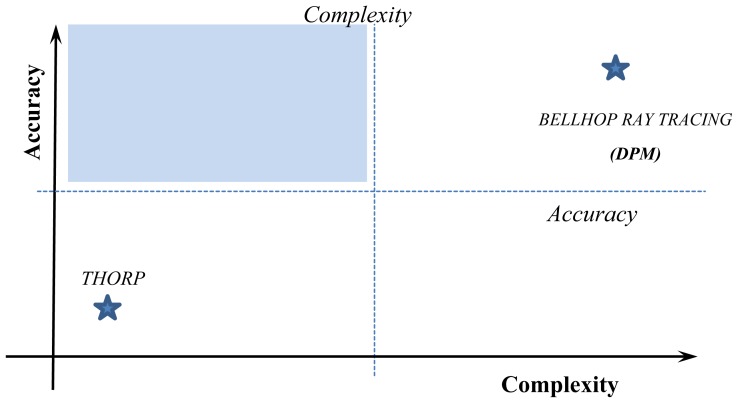
Tradeoff between model propagation accuracy and computational complexity.

Tradeoff between model complexity and accuracy is shown in Figure 2, where accuracy and complexity thresholds are defined. The shaded area covers those propagation models with the minimum acceptable model propagation accuracy that leads to reliable prediction results and, at the same time, low computational complexity overhead to perform detailed and scalable network simulations.

The rest of this work is organized as follows: in Section 2 we define a specific network scenario and discuss the computational demands of deterministic propagation models. The statistical propagation model we propose is described in Section 3. In Section 4 we discuss the implications that statistical modeling can have on network planning. Finally, in Section 5 some conclusions are drawn.

## 2. System Set Up

Now we are going to define the overall system where we have developed our study. First, we will define the geographical location and dimensions of the network scenario, including the environmental parameters like bathymetry, floor sediment composition, sound speed profile, water temperature and surface wave activity, among others, that could be found in global ocean databases [13–15]. Then, the network specific parameters are defined, like network topology, number of nodes, acoustic signal frequency and transmission range. In order to evaluate the impact of node movement in the acoustic signal propagation, we have described a simple model that represents the random movement of network nodes anchored to the floor, mainly due to marine currents or tides.

**Figure 3.**
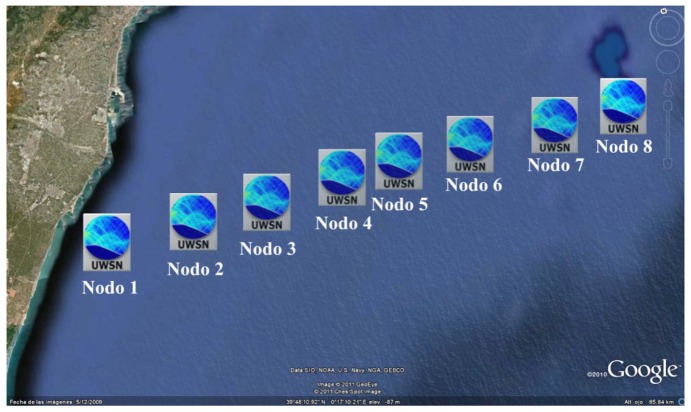
Network deployment in Valencia, Spain.

The network of interest is located in coastal waters near Valencia, Spain, at coordinates 39°48′13.14″N and 0°4′34.53″W. It consists of eight nodes arranged in a linear topology, as illustrated in Figure 3.

For our purposes, the source is assumed to be at one end (closest node to the coastline), and the rest of the nodes are placed at different distances ranging from 500 m to 3,700 m. All nodes are anchored to the sea floor in such a way that their depth is 10 meters, while the water depth varies from 25 m to 35 m within the network scenario of 5,000 × 5,000 m^2^. Should we wish to employ a different network scenario, the procedure would be the same, since all network scenario and environmental parameters could be obtained from on-line global databases, and the rest of parameters may be fixed in our simulation framework. We assume a fixed network topology, and vary the parameters related to the surface wave activity (wave height and wave length). The surface parameters are taken from historical and prediction values from National Geophysical Data Center databases [16,17].

We also account for the fact that an acoustic communication signal does not consist of a single frequency, but occupies a (possibly wide) certain bandwidth as a result of the acoustic signal modulation employed. The overall transmission loss is computed along the whole network scenario by running the DPM with the Bellhop approach. Each DPM simulation run produces the acoustic field values in a 5 km × 5 km × 30 m volume, with a resolution of 0.33 m^3^. The values corresponding to receiver node locations are then extracted, and a statistical analysis is performed for each position.

To compute the transmission loss, we have used two different approaches: (1) assuming single frequency acoustic signals, where several experiments were performed with frequency ranging from 5 to 80 kHz; and (2) assuming a more realistic approach, taking as reference the Evologics Modems technical data sheet [18] to choose the center frequency and the bandwidth of three different frequency bands, a low-frequency band of 5–15 kHz (S2C R 8/16 modem), a mid-frequency band of 20–34 kHz (S2C R 18/34) and the high-frequency one of 50–75 kHz.

**Figure 4.**
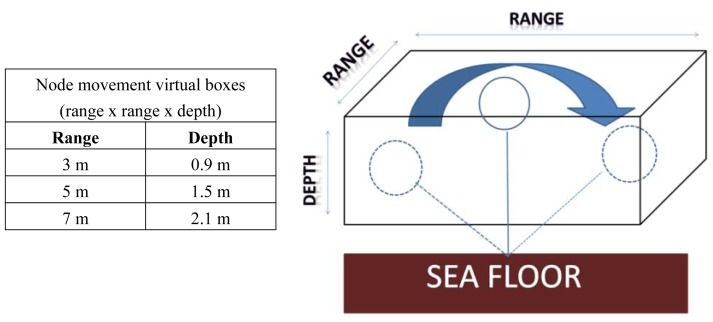
Network node movement model.

Although the network topology is fixed, *i.e.*, node position is always the same, we have considered, as explained before, that all the network nodes have a random oscillatory mobility (typically larger in horizontal than in vertical direction) due to the nature of the underwater environment and the anchor system. The movement is typically slow and constrained to a specific water volume around the reference placement location. In order to simplify the proposed node mobility model, we will consider that the anchored node may be at whatever point inside the virtual box of dimensions Range x Range x Depth as shown in Figure 4.

For each experiment, all network nodes employ the same power transmission. Table 1 summarizes the fixed and variable system parameters used in the simulation experiment.

The hardware used to run all the simulations is a cluster of computers that consist of six nodes, each one with four CPUs of 1 GHz and 8 GB of RAM, for a total of 24 cores, all governed by Rocks Cluster OS version 4.3 [19] and using Condor Project software version 6.8.5 [20] to manage the parallel DPM model simulations.

Each execution of the Bellhop tool [9] takes about 5 minutes on a single CPU. Considering 14 different wave heights and 14 different wave lengths, *i.e.*, 196 different scenarios, and 56 different frequencies (5–15, 20–34, 50–75, 35, 40, 45 and 80 kHz), a total of more than 10,000 simulations were performed, taking around 40 hours of computation time to obtain all the data used for our statistical analysis.

## 3. Statistical Prediction Model

We have introduced the fact that an ensemble of transmission loss values, obtained by varying the physical conditions along a range of frequencies, obeys a log-normal distribution. The statistical model proposal is an attempt to replace this heavy computational process with a simple expression that offers transmission loss predictions as reliable as the propagation model. The study of the log-normal distribution requires focusing on both the parameters required to build the distribution, the mean (*μ*) and standard deviation (*σ*). These parameters will depend on the distance, *d*, and the acoustic signal frequency, *f*:

(1) SPM(d,f)=μ(d,f)+σ(d,f)

### 3.1. Mean Value

The study of the mean value of the Equation (1) requires an accurate process to calculate the expression as it is going to be the base value within the whole formula. So, we will proceed to statistically predict the results of the DPM model, taking into account a range of signal frequencies from 5 to 80 kHz in steps of 5, each of them combined with 196 different surfaces working under the selected scenario with the parameters found in Table 1.

In order to estimate the mean transmission loss, we have employed the Surface Fitting Tool from MATLAB R2011a [21]. The parameters employed to perform the surface fitting with a polynomial approximation are distance and frequency. In order to get a good fitting with a low complexity formula, we established the distance (d) as a quadratic variable and frequency (f) as a lineal one.

Achieving a coefficient of determination (R2) of 0.96, the single frequency mean transmission loss, sfμ(d,f), is obtained with the resulting fitting Equation (2):

(2) $$sf\mu (d,f)=k_{1}d^{2}+k_{2}d+k_{3}df+k_{4}f+k_{5}$$

where *k_1_* = −0.0000012, *k_2_* = 0.007766, *k_3_* = 0.0002786, *k_4_* = 0.0332, *k_5_* = 36.6.

**Figure 5.**
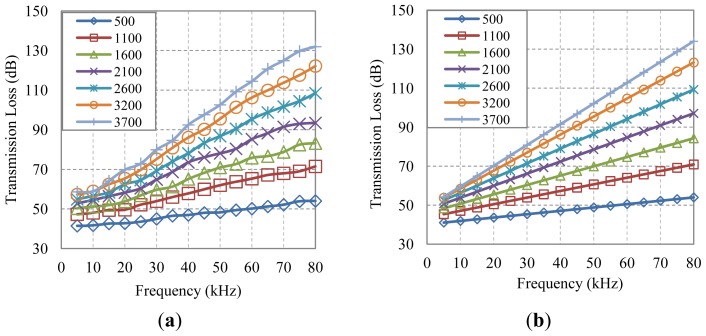
Average transmission loss *vs.* frequency; (**a**) Bellhop, and (**b**) SPM mean.

In Figure 5, we show the plots representing the average transmission loss (sfμ) at different values of frequency and distance supplied by: (a) the Bellhop model, and (b) the statistical prediction model (SPM) defined in Equation (2). In order to determine the introduced error, we calculate the average error of all frequencies at a particular distance, being always less than 1.6 dB.

Since real implementations perform signal modulations that produce a specific bandwidth, not a single frequency, we proceed to extend the single frequency SPM model defined above to acoustic signals with a particular bandwidth. The process to obtain the average transmission loss (signal attenuation values) out of a range of frequencies is done as follows: for each spatial position in the network scenario, we calculate the inverse of the attenuation values for each single frequency composing the desired bandwidth, and then we obtain their average. In Equation (3) we define the general expression and an example for a bandwidth of 5–15 kHz (composed by 11 single frequencies spaced at 1 kHz) to calculate the overall attenuation (A):

(3) $$\begin{array}{c} \frac{1}{A_{R}}=\left[\frac{1}{A_{1}}+\frac{1}{A_{2}}+\frac{1}{A_{3}}+\frac{1}{A_{4}}+\cdots +\frac{1}{A_{N}}\right]/N\\ \frac{1}{A_{5-15}}=\left[\frac{1}{A_{5}}+\frac{1}{A_{6}}+\frac{1}{A_{7}}+\frac{1}{A_{8}}+\cdots +\frac{1}{A_{15}}\right]/11 \end{array}$$

Now, the transmission loss corresponding with the three frequency bands are plotted together with the transmission loss of their central frequencies calculated with Equation (2). At 5–15 kHz we have a bandwidth of 11 frequencies, at 20–34 kHz there are 14 frequencies and 25 in the 50–75 kHz band. So, for each one we select their corresponding central frequencies of 10, 27 and 62.5 kHz, respectively.

**Figure 6.**
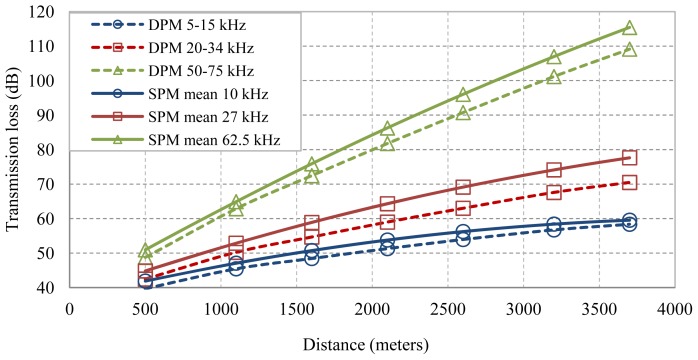
Attenuation of bandwidth signals with DPM and SPM single frequency proposal.

Figure 6 let us find out that the SPM single frequency proposal is not valid for bandwidth signals to properly estimate the transmission loss by means of Equation (2). The set of frequencies 5–15, 20–34 and 50–75 kHz are always below the ones obtained by the SPM approach with the single frequencies 10, 27 and 62.5 kHz. So, in order to reduce the estimation error we will define a bandwidth correction factor, *bcf(d,b)*, using the distance (d) and bandwidth (b) as parameters. The fitting of the bandwidth correction factor is divided into two expressions: a Fourier fitting for bandwidth below 14 kHz with an R2 of 0.74, and a polynomial fitting of degree one for bandwidth over 14 kHz, resulting in the Equation (4). Then, the estimation of the mean transmission loss (5) will be composed by two terms, one for single frequency (b = 1) and the other for a center frequency, *f*, with a bandwidth *b*:

(4) $$\text{bcf}(d,b)+\{\begin{array}{c} k_{6}+k_{7}\cos(d*k_{8})+k_{9}\sin(d*k_{8}),b<14\\ k_{10}+k_{11}b+k_{12}d,b\geq 14 \end{array}$$

where *k_6_* = 2.076, *k_7_* =0.4811, *k_8_* = 0.002528, *k_9_* = −0.2722, *k_10_* = 2.547, *k_11_* = −0.06234, *k_12_* = 0.001532:

(5) $$\mu (d,f)=\{\begin{array}{r} sfu(d,f),b=1\\ sfu(d,f)-bcf(b),b>1 \end{array}$$

**Figure 7.**
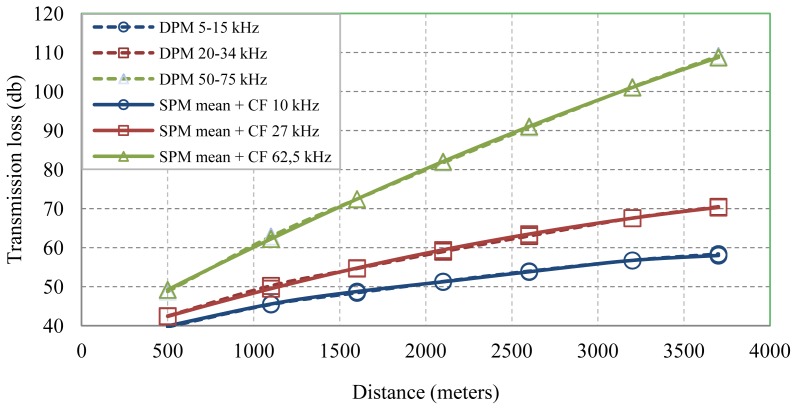
Bandwidth signals loss with DPM and SPM single frequency proposal after applying the bandwidth correction factor.

Applying the bandwidth correction factor to the SPM single frequency approach, the estimation error is considerably reduced, as it can be seen at Figure 7. So, by using Equation (5), we can proceed to perform accurate estimations of both single frequency and bandwidth signals.

It is time now to analyze the impact on the transmission loss of the node movement defined in our target scenario, where three different node movement models have been defined. These movement models are parameterized with depth and range with a bandwidth signal of 5–15 kHz (center frequency 10 and bandwidth 11 kHz) at every node position in the scenario.

**Figure 8.**
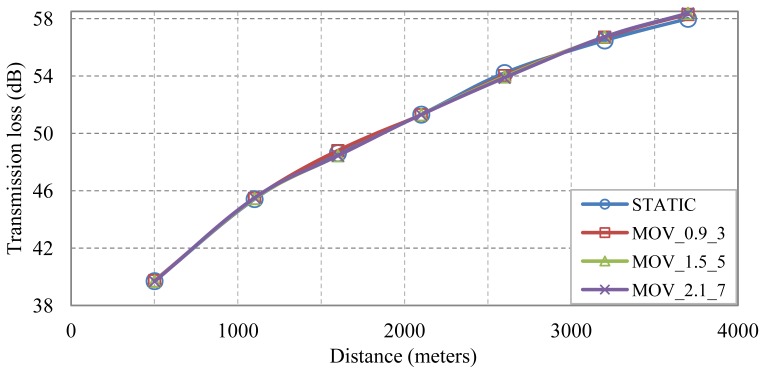
Attenuation *vs.* distance with different node movement at 5-15 kHz bandwidth signals.

In Figure 8 we compare the static (no node movement) DPM results *vs.* the ones including the node movement. As it can be seen, the node movement has no effect on the mean value, where the average and maximum differences are 0.07 and 0.38 dB, respectively. The same behavior happens at the other frequencies; at 20–34 kHz, the average and maximum differences are 0.08 and 0.53, respectively; and at 50–75 kHz, the average difference is 0.1 dB and the maximum 0.49 dB. So, we may consider that the node movement defined in our scenario has no impact on the estimation of the mean attenuation value.

### 3.2. Standard Deviation Value

**Figure 9.**
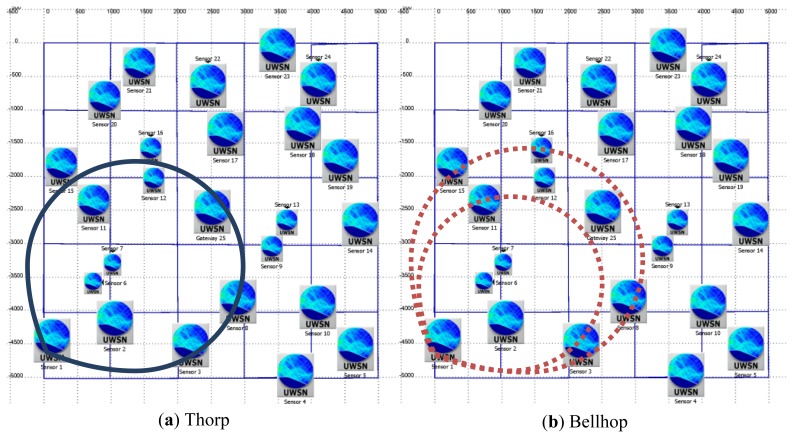
Gateway reachability (central node) from Node #1 (bottom leftmost node).

The study of the Standard Deviation Value (σ) is essential to obtain a statistical expression that would accurately describe the behavior shown in Figure 1. The objective is that the proposed expression experiences the same variability around the nominal (mean) attenuation value found at a particular spatial location inside the network scenario. This would lead to more realistic attenuation predictions that are caused by environmental parameters like surface wave activity. In Figure 9, a network scenario with 25 network nodes is shown. If a simple prediction model is used, like Thorp's model where the transmission loss is dependent on frequency and distance [22], the transmission range for node #1 (located at the lower left corner) will be always the same-represented by the solid-line circle in Figure 9(a) and, as a consequence, the reachable neighbors will be the same during the entire simulation time. However, in Figure 9(b) when the Bellhop propagation model is used under a scenario with environmental varying conditions, the effective transmission range is variable-represented by the disjointed area of the two dashed-line circles—and the reachable neighborhood is also variable during the entire simulation time. Thus, our statistical approach needs to represent the same variability found in the Bellhop model, being very important to estimate the proper standard deviation value, σ, so the reachability to other nodes will change during the simulation with a similar distribution as the one found with the Bellhop model.

**Figure 10.**
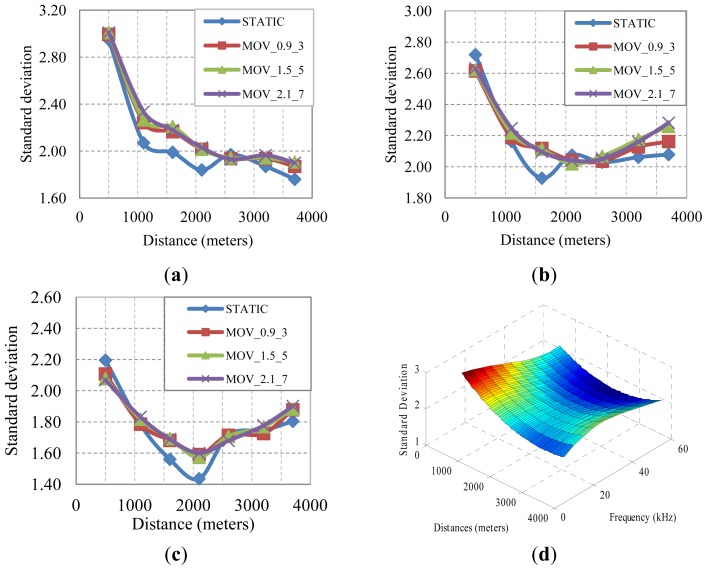
Standard deviation of attenuation *vs.* distance with different node movement approaches and frequency ranges. (**a**) 5–15 kHz, (**b**) 20–34 kHz, (**c**) 50–75 kHz, (**d**) Node movement 1,5 meters height, 5 meters range.

In order to study σ, we will use the same bandwidth signals as the ones used in the previous section, 5–15 kHz, 20–34 kHz and 50–75 kHz, as well as the same node movement model described in Figure 4. We have run the Bellhop model with this new network scenario, the parameters found in Table 1, and the node movement model described earlier, to obtain the evolution of standard deviation values as a function of the distance. The different curves represented in Figure 10(a,b,c) correspond with the static node approach and the three node movement configurations described earlier for acoustic signal bands of 5–15 kHz, 20–34 kHz, and 50–75 kHz, respectively.

As it can be seen at Figure 10, all the node movement configurations exhibit almost identical behavior, where a bigger σ value is found at distances below 1,000 m, and for farther distances the σ oscillates from 1.76 to 2.3. In Figure 10(d) we show a 3D graph of one of the node movement configurations (MOV_1.5_5: 1.5 m depth and 5 m range) that represents the standard deviation as a function of distance and frequency. There is a higher difference at 500 meters and, as commented earlier, the remaining values are within a 1.7 and 2.3 (a difference of 0.6) range. After testing several regression approaches to obtain the corresponding surface fitting that estimates the standard deviation value, we have performed the polynomial approach represented in Equation (6) where *d* and *f* represent distance and frequency values, respectively. The fitting accuracy is represented with a R2 of 0.9804:

(6) $$\sigma (d,f)=k_{1}d^{3}+k_{2}d^{2}f+k_{3}f^{2}d+k_{4}d^{2}+k_{5}df+k_{6}f^{2}+k_{7}d+k_{8}f+k_{9}$$

where *k_1_* = −0.06468, *k_2_* = −0.01726, *k_3_* = −0.1214, *k_4_* = 0.1794, *k_5_* = 0.1477, *k_6_* = −0.1277, *k_7_* = 0.07606, *k_8_* = −0.116, k_9_ = 2.013.

Finally, we have determined the mean and standard deviation values of a log-normal distribution that properly represents the same behavior as the Bellhop acoustic propagation model, taking into account the transmission loss variability induced by environmental scenario parameters, and the node movement typically found in underwater deployments.

## 4. Implication for Network Planning

The apparent match between the results of deterministic and statistical models motivates the SPM use for network design and analysis via simulation. Consider, for example, network simulation over a prolonged interval of time that spans varying propagation conditions and involves the transmission of a large number of data packets over multiple hops. If deterministic modeling is used, each packet transmission requires one execution of the Bellhop ray tracer, which soon becomes excessively long for a growing number of data packets (assuming 5 minutes for each Bellhop run and a single frequency, simulation of 100,000 packets would take about a year). Although the DPM offers an accurate solution for the particular geometry observed at any given moment in time, its execution makes the simulation times unaffordable for the benchmarking and testing of the upper layer protocols.

In contrast, a statistical model can take several hours to compute (40 hours in the example we have presented) a particular network scenario, but this would be needed only once for a network scenario. After that, for a particular simulation run, each packet transmission only requires a single call to a Gaussian random number generator to determine the transmission loss. Moreover, if network topology changes slightly, or if a new node is added, the statistical model needs to be augmented only by the corresponding set of nominal parameters (mean and standard deviation for the newly created links).

**Figure 11.**
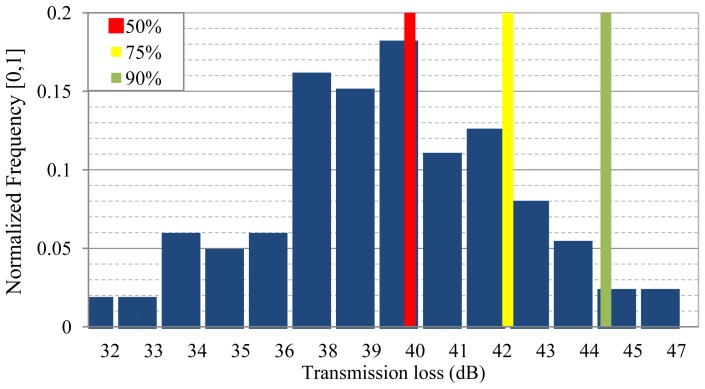
Transmission loss normalized histogram of an ensemble of channel realizations.

Most important, the statistical model can easily be used to assess transmit power allocation that will guarantee successful data packet reception with a desired level of performance (e.g., link reliability).That is to say, the proposed SPM can easily be used to calculate the transmission loss values that are not exceeded with a given probability. For example, a 90% transmission loss would be that value which is not exceeded for 90% of the time, *i.e.*, in 90% of channel realizations. In Figure 11 we show the normalized histogram obtained from an ensemble of channel realizations corresponding to varying propagation conditions in a given network scenario.

We have highlighted three link reliability levels, corresponding to successful channel realization probabilities of 50%, 75%, and 90%. This information will assess the transmission power required to guarantee the destination node reachability with a specific probability.

**Figure 12.**
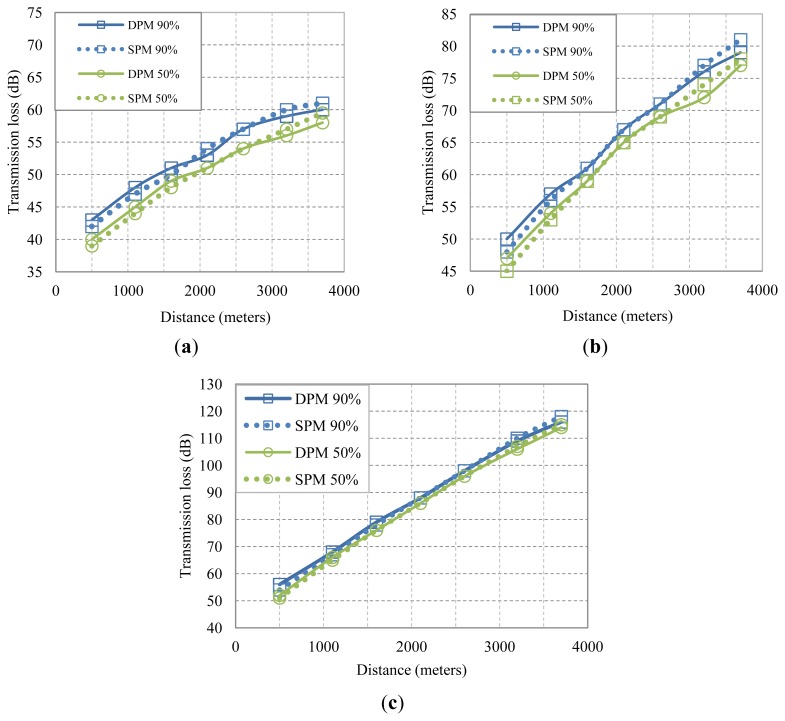
The transmission *vs.* distance curves with different power allocation schemes (50%, 90%). The solid and dashed curves show the results from the deterministic and the statistical propagation models, respectively. (**a**) 5–15 kHz, (**b**) 20–34 kHz, (**c**) 50–75 kHz.

Figure 12, shows 50% and 90% transmission loss for our example system. We observe a good match between the values predicted by the deterministic model and those of the statistical model. Note that the X% values of the SPM are computed analytically, based only on the knowledge of the mean and standard deviation.

The availability of X% values is significant for determining the transmit power necessary to achieve a certain level of performance. Typically, network planning is based on the nominal ray trace, *i.e.*, on the 50% transmission loss to which some margin may be added. If transmit power allocation is based on a different value, say 90% transmission loss instead of the nominal 50%, data packets will be more likely to reach their destinations. More power will be needed at the same time, but the overall network performance may improve. We say may improve, because a higher transmit power also implies higher levels of interference. The resulting performance trade-offs are generally hard to address analytically, and are instead assessed via simulation. A statistical propagation model that directly links the transmit power to the X% transmission loss then becomes a meaningful and useful tool for system design.

## 5. Conclusions

Large-scale design of an underwater acoustic network requires a judicious allocation of the transmit power across different links, to ensure a desired level of system performance (connectivity, throughput, reliability, *etc.*). Because of the inherent system complexity, simulation analyses are normally conducted to assess the performance of candidate protocols under different resource allocation policies. These analyses are often restricted to the use of deterministic propagation models, which, although accurate, do not reflect the random time-varying nature of the channel.

While, in principle, it is possible to examine the network performance for a large set of perturbed propagation conditions, the computational complexity involved in doing so is extremely high. To facilitate network simulation in the presence of channel fading, we investigated a statistical modeling approach. Our approach is based on establishing the nominal system parameters for a desired deployment location (water depth, sediment composition, operational frequency range) and using ray tracing to compute an ensemble of transmission losses for typical inter-node distances. An ensemble is generated by considering a set of perturbed surface conditions, defined by varying wave activity (height, period). The so-obtained ensemble is then used to determine the statistical parameters of a hypothesized log-normal distribution of the transmission loss. For a representative example of a small network operating in a 5 km × 5 km area with inter-node distances ranging between 500 m and 4 km, it was found that the mean can be well approximated as a linear function of the logarithm of distance, while the variance can be modeled as constant over given ranges of distances. More elaborate and more accurate models than the lognormal one can also be developed using this approach.

A statistical model of this type enables computationally-efficient inclusion of fading effects into a network simulator. Namely, to assess the average system performance, network operation has to be simulated over a large set of channel realizations (e.g., varying surface conditions). Whereas repeated computation of the ray trace for different hops traversed by each of the data packets in a given network may be computationally prohibitive, statistical modeling requires only a single call to the Gaussian random generator for each packet transmission. The overall simulation time is thus considerably reduced, allowing a system designer to freely experiment with varying protocols and resource allocation strategies in an efficient manner. The ultimate goal of such computational experiments is to choose the best upper-layer protocol suite and relate the necessary system resources (power, bandwidth) to the propagation conditions, *i.e.*, to the statistical parameters of the transmission loss (e.g., X% value), which can in turn be easily generated using the proposed method of statistical modeling.

## Figures and Tables

**Table 1.** System Parameters.

**Parameter**	**Value**
Transmission range	500 m to 3,700 m (in steps of ∼500 m)
Scenario Area	5,000 m × 5,000 m
Sediment floor	Gravel
Month	August
Wave height (m)	1 m to 3 m (in steps of 0.15 m)
Wave length (m)	100 m to 150 m (in steps of 3.5 m)
Frequency (kHz)	5 to 80 kHz (in steps of 5 kHz) (5–15)(20–34)(50–75) kHz (in steps of 1 kHz)
Scenario depth (m)	25–35
Global load (packets/s)	5
Data packet size (bits)	1,024
Control packet size (bits)	24
Simulation time (s)	3,600
